# Comparison of Baseline and Post-Nitrate Exercise Testing in Patients with Angina but Non-Obstructed Coronary Arteries with Different Acetylcholine Test Results

**DOI:** 10.3390/jcm13082181

**Published:** 2024-04-10

**Authors:** Angelo Giuseppe Marino, Giuseppe Gentile, Ludovica Lenci, Fabio De Benedetto, Saverio Tremamunno, Nello Cambise, Antonietta Belmusto, Antonio Di Renzo, Lorenzo Tinti, Antonio De Vita, Gaetano Antonio Lanza

**Affiliations:** 1 Dipartimento di Scienze Cardiovascolari e del Torace, Università Cattolica del Sacro Cuore, 00168 Rome, Italy; 2 Fondazione Policlinico Universitario A. Gemelli IRCCS, 00168 Rome, Italy; antonio.devita90@gmail.com

**Keywords:** angina syndromes, non-obstructive coronary artery disease, exercise stress test, acetylcholine test, nitrates

## Abstract

**Background:** Intracoronary acetylcholine testing may induce epicardial coronary artery spasm (CAS) or coronary microvascular spasm (CMVS) in patients with angina syndromes but non-obstructive coronary artery disease, but their causal role in individual patients is not always clear. In this prospective, observational single-center study, we aimed to assess whether (1) the induction of myocardial ischemia/angina by electrocardiogram (ECG) exercise stress test (EST) differs between patients showing different results in response to acetylcholine testing (i.e., CAS, CMVS, or no spasm); (2) the preventive administration of short-acting nitrates has any different effects on the EST of those patients who showed a positive basal EST. We expected that if exercise-induced angina and/or ischemic ECG changes are related to CAS, they should improve after nitrates administration, whereas they should not significantly improve if they are caused by CMVS. **Methods:** We enrolled 81 patients with angina syndromes and non-obstructive coronary artery disease, who were divided into three groups according to acetylcholine testing: 40 patients with CAS (CAS-group), 14 with CMVS (CMVS-groups), and 27 with a negative test (NEG-group). All patients underwent a basal EST (B-EST). Patients with a positive B-EST repeated the test 24–48 h later, 5 min after the administration of short-acting nitrates (N-EST). **Results:** There were no significant differences among the groups in terms of the B-EST results. B-EST was positive in eight (20%) patients in the CAS-group, seven (50%) in the CMVS-group, and six (22%) in the NEG-group (*p* = 0.076). N-EST, performed in eight, six, and five of these patients, also showed similar results in the three groups. Furthermore, the N-EST results also did not significantly differ compared to B-EST in any group, remaining positive in seven (87.5%), four (66.7%), and four (80%) patients in the CAS-group, CMVS-group, and NEG-group, respectively (*p* = 0.78). **Conclusions:** Our data show that patients with angina and non-obstructive coronary artery disease show largely comparable results of the ECG exercise stress test and similar poor effects of short-acting nitrates on abnormal ECG exercise stress test results. On the whole, our findings suggest caution in attributing to the results of Ach testing a definite causal role for the clinical syndrome in individual patients.

## 1. Introduction

Up to 50–60% of patients with episodes of chest pain suggesting stable angina and about 15% of patients admitted to hospital with a clinical diagnosis of non-ST-segment elevation acute coronary syndrome (NSTE-ACS) are found to have non-obstructive coronary artery disease (NO-CAD) at angiography [1,2,3]. 

In a proportion of both groups, the intracoronary acetylcholine (Ach) provocation test induces epicardial coronary artery spasm (CAS) or coronary microvascular spasm (CMVS) [4,5,6]. Furthermore, impaired coronary microvascular dilatation has been reported in a relevant proportion of these patients [7,8,9]. However, although the detection of these findings gives evidence for the presence of abnormalities in coronary epicardial function and/or microcirculation, the role that the single alterations play in the clinical syndrome of individual patients might not always be clear. Specifically, in some patients with angina and NO-CAD, the induction of CAS by Ach during angiography might erroneously lead to attributing their angina pain to this mechanism, when symptoms are instead caused by CMVS. Ach in these patients might, indeed, induce both CAS (just as an expression of coronary hyper-reactivity) and CMVS, but the latter will inevitably go undetected due to the presence of CAS. Thus, in these cases, the true mechanism responsible for the clinical picture of the patient (i.e., CMVS) will be missed [3]. 

The electrocardiogram (ECG) exercise stress test (EST) may induce ischemic ST-segment changes and/or typical chest pain in patients with angina syndromes and NO-CAD [10,11,12]. However, it is not clear whether the results of ESG-EST may help distinguish among the different possible mechanisms responsible for angina symptoms in this heterogeneous population of patients. Specifically, it is not clear whether, in patients showing CAS induction by Ach testing, ischemic symptoms and/or ECG changes induced by an EST can actually be related to exercise-induced CAS rather than a missed CMVS, or even an impaired coronary microvascular dilatation. 

Previous studies have consistently shown that CAS is highly responsive to, and can effectively be prevented by, short-acting nitrate administration [13], whereas both CMVS and impaired coronary microvascular dilatation present an inconsistent response to short-acting nitrates [14,15,16]. Accordingly, we hypothesized that if, among patients with chest pain and NO-CAD, EST-induced angina and/or ischemic ECG changes are related to CAS, they should be prevented or significantly improve when the EST is performed after the administration of short-acting nitrates. In contrast, such an improvement might not occur if EST-induced angina and ischemic ECG changes are caused by CMVS or reduced vasodilatation [14,15,16].

Thus, the aims of this prospective, observational, single-center study were twofold: (1) to assess whether, among patients with angina and NO-CAD, ECG-EST results differ between groups showing different results on Ach testing; (2) to assess whether the preventive administration of short-acting nitrates has any different effects on the ECG-EST results of the different Ach test groups, thus helping identifying the pathophysiologic mechanisms responsible for the clinical picture of individual patients. 

## 2. Materials and Methods

### 2.1. Patients

From January 2021 to June 2023, a total of 343 consecutive patients undergoing coronary angiography at our hospital because of chest pain suspected for angina were found to have NO-CAD and were considered for the study. Eighty-one patients (23.6%) who fulfilled the following inclusion criteria were eventually included in the study: (1) episodes of stable, exercise-induced chest pain compatible with typical angina or admission to our hospital with a clinical picture of NSTE-ACS, i.e., chest pain at rest with ST-segment depression and/or T wave changes at the ECG, with or without a typical rise and fall of troponin I serum levels; (2) no significant stenosis (>50% reduction in epicardial lumen and/or fractional flow reserve <0.80) at coronary angiography; (3) Ach test performed during coronary angiography.

Patients (n = 262) were excluded from the study because of the presence of one or more of the following exclusion criteria: (1) conditions that could preclude a symptom-limited EST; (2) ECG abnormalities that could limit ST-segment analysis during the EST, e.g., non-sinus rhythm, conduction disorders, significant basal ST-segment/T wave changes; (3) history of specific heart disease, e.g., ischemic (previous surgical or percutaneous myocardial revascularization), valvular, congenital heart disease or cardiomyopathy; and (4) lack of functional tests during invasive investigation. All patients gave their written informed consent to participate in the study, which was part of a larger study involving patients with angina and normal coronary arteries, which was approved by the Ethics Committee of Fondazione Policlinico Universitario A. Gemelli IRCCS, Università Cattolica del Sacro Cuore, Rome, Italy (PRECISION study; Prot. 36077/19; ID 2747). 

### 2.2. Acetylcholine Test

The Ach test was performed during invasive coronary angiography by the intracoronary infusion of ACh into the left coronary artery at increasing doses of 20, 50, and 100 μg over a period of 3 min each, and with a 3 min interval between injections. Coronary angiography was performed at the end of each dose, or immediately in the case of the induction of chest pain and/or ischemic ECG changes. The test was interrupted in the case of positive findings or the occurrence of any side effect. In the case of a negative test in the left coronary artery, the test was also performed in the right coronary artery, by administering doses of 20 and 50 µg only.

An epicardial spasm was diagnosed in the case of focal or diffuse narrowing of the coronary diameter ≥90%, associated with typical symptoms and/or ischemic ECG changes. CMVS was diagnosed when typical symptoms and/or ischemic ECG changes were induced in the absence of epicardial spasm at angiography, as defined above. 

Based on the result of the Ach test, patients were divided into three groups: (1) patients with the induction of epicardial coronary artery spasm (CAS-group); (2) patients with the induction of coronary microvascular spasm (CMVS-group); and (3) patients with a negative test (NEG-group).

### 2.3. Exercise Stress Test

All patients underwent a basal EST (B-EST) after the withdrawal of anti-ischemic drug therapy for at least 5 half-lives. All ESTs were performed in the early afternoon following the standard treadmill Bruce protocol. Three ECG leads (V2, aVF, and V5) were continuously monitored during the test. A standard 12-lead ECG was printed, and blood pressure was measured by a cuff sphygmomanometer at the onset of the test, at the end of each stage, and at peak exercise, as well as at 1 mm ST-segment depression (STD), when angina occurred, and when it was clinically indicated. Several 12-lead ECG strips were printed when STD started to appear on the screen in order to time exactly when 1 mm STD occurred. 

EST was terminated when one or more of the following endpoints were achieved: (1) physical exhaustion; (2) progressive angina (Borg scale > 6); (3) STD > 4 mm; and (4) occurrence of potentially harmful clinical conditions (hypotension, severe arrhythmias, worsening dyspnea). 

The test was considered positive for myocardial ischemia when a horizontal or down-sloping STD of >1 mm was observed in at least one lead. The ECG strips of both tests were blindly and independently evaluated by two expert cardiologists. Discrepancies were resolved by consensus, under the supervision of a third expert cardiologist. 

Patients who showed significant ischemic ST-segment changes and/or developed typical chest pain at the B-EST underwent a second EST 48–72 h after the baseline test, after the administration of 5 mg of sublingual isosorbide dinitrate (nitrate-EST, N-EST) to assess the potential response to vasodilator therapy. The drug was given 5 min before starting the EST.

The tests were performed following the same procedure and criteria of the B-EST.

For each EST, we obtained heart rate (HR), systolic and diastolic blood pressure (BP), and rate pressure product (RPP = HR × systolic BP) at baseline, 1 mm STD, angina, and peak exercise, as well as time to 1 mm STD, angina, and peak exercise. Maximal STD was also recorded. For statistical analyses, the values at peak exercise were used for those at 1 mm STD or angina when STD and/or angina did not occur during the second exercise test (N-EST).

### 2.4. Statistics

Baseline continuous variables between groups were compared by analysis of variance (ANOVA), whereas discrete variables were compared by the chi-square test or Fisher’s exact test, as indicated. The comparisons of the changes in continuous variables at N-EST vs. B-EST within the 3 groups were performed by a generalized linear model with a repeated measure design. An unpaired and paired t-test was applied for multiple comparisons between and within groups, respectively, in the case of a global statistical significant difference among the groups. A *p* < 0.05 was required for statistical significance. Variables are reported as mean ± SD or proportions. The SPSS 28.0 statistical software (SPS Inc., Florence, Italy) was used for the analysis of the data. 

## 3. Results

### 3.1. Patients

Overall, 81 patients were included in the study: 40 patients (49%) in the CAS-group, 14 patients (17%) in the CMVS-group, and 27 patients (34%) in the NEG-group. The main clinical characteristics of the three groups of patients are summarized in Table 1. There were no statistically significant differences among the groups in terms of age, sex, cardiovascular risk factor, and clinical presentation, i.e., stable angina, unstable angina, or myocardial infarction with non-obstructed coronary arteries (MINOCA). A higher proportion of patients was taking calcium-channel blockers in the CAS-group (95% vs. 71% in the CMVS-group and 26% in the NEG-group; *p* < 0.001), whereas beta-blockers were more frequently used in the NEG-group and CMVS-group compared to the CAS-group (37%, 29%, and 13%, respectively; *p* = 0.01). Statin therapy was taken by a high proportion of patients in the CAS-group (78%) and NEG-group (67%), but in 36% of the CMVS-group patients only (*p* = 0.02).

### 3.2. Basal Exercise Stress Test

The main results of the B-EST are summarized in Table 2. There were no significant differences in the EST parameters among the groups, although the NEG-group tended to show a longer EST duration (*p* = 0.06). Overall, 21 patients only (25.9%) showed a positive B-EST. There was a tendency for a higher prevalence of positive EST in the CMVS-group (7 patients, 50%) compared to the CAS-group (8 patients, 20%) and NEG-group (6 patients, 22%), but the differences did not achieve statistical significance (*p* = 0.076). Angina was induced in 10 patients only, with a higher prevalence in the CMVS-group (4 patients, 28%) than in the CAS-group (4 patients, 10%) and NEG-group (2 patients, 7%), but the difference did not achieve statistical significance (*p* = 0.08).

### 3.3. B-EST vs. N-EST

Of 21 patients who showed a positive B-EST, 2 (9.5%) refused to undergo N-EST (1 in CMVS-group and 1 in NEG-group). Thus, N-EST was performed in 19 patients, i.e., 8, 6, and 5 patients in the CAS-, CMVS-, and NEG-groups, respectively. 

Detailed results of the ESTs of patients who completed both the B-EST and the N-EST are shown in Table 3. The results of the N-EST did not differ significantly among these three groups of patients. The EST remained positive for ST-segment depression in seven (87.5%) patients in the CAS-group, in four (66.7%) patients in the CMVS-group, and in four (80%) patients in the NEG-group (*p* = 0.64). No significant differences were also observed in the induction of angina pain among groups and between the two ESTs (Table 3).

## 4. Discussion

The most relevant findings of the present study are as follows: (1) the ECG-EST results were comparable in patients with angina syndromes with NO-CAD with a different response to Ach testing; (2) in patients with a positive ECG-EST, the preventive administration of short-acting nitrates was, on the whole, unable to normalize or significantly improve the results of ECG-EST, independently of the results of Ach provocation testing.

Some recent guidelines do not recommend the use of ECG-EST as a reference test to detect obstructive CAD [17,18] because of the higher sensitivity of noninvasive stress imaging modalities, coronary computed tomography angiography and functional assessment of coronary stenoses, as well as the perceived high false positive rate of the ECG-EST [19,20,21]. However, myocardial ischemia can occur because of CAS, CMVS, or coronary microvascular dysfunction (CMD) in the absence of obstructive CAD. In this regard, a recent study challenges the traditional belief that the EST has a high false positive rate, suggesting instead a 100% specificity in identifying CMD [22]. In our study, we aim to assess whether the EST can also play a role in helping to identify the mechanism responsible for clinical syndrome in patients with angina and NO-CAD.

Coronary microvascular dysfunction has for a long time been considered a major cause of angina chest pain in patients with NO-CAD [3,23,24]. Several studies, however, showed that a consistent group of these patients develops CAS during Ach testing, suggesting that this mechanism might more frequently be involved than what was previously believed [4,5,6,25,26]. The fact that Ach induces CAS in a proportion of these patients, however, does not necessarily reflect a causal relation with the clinical symptoms of the patient, which might in fact be determined by some other mechanism, specifically coronary microvascular constriction/CMVS or even abnormal coronary microvascular dilation. 

This possibility is suggested by several observations. First, while one would expect a higher proportion of Ach-induced CAS in patients with an acute presentation or rest angina, the proportion of patients developing Ach-induced CAS is rather similar independently of the clinical presentation. For example, in a large study by Ong et al. including >900 patients undergoing an Ach test, CAS was induced in 34% of patients with rest angina and in 30% of those with effort angina [4]. This finding was confirmed in the present study, in which CAS was induced by Ach in 15 out of 34 patients with a history of stable (exercise-induced) angina (44%) and in 25 out of 47 patients admitted with chest pain at rest indicating an NSTE-ACS (53%; *p* = 0.44).

Second, in two studies, Ach induced chest pain/ECG changes in patients with angina and NO-CAD before the induction of CAS in 17% and 33% of patients, respectively [27,28], suggesting that CMVS can be induced together with the occurrence of CAS and might actually be responsible for the clinical symptoms in at least a proportion of patients.

Third, in a recent randomized study, the calcium-channel blocker diltiazem failed to improve symptoms in patients with Ach-induced CAS, and the reduction in CAS induction by diltiazem during a follow-up Ach test was associated with greater evidence of CMVS [29]. Of note, the published results of a recent randomized controlled trial failed to show favorable effects on the angina status of invasive-provocative-test-guided medical therapy in patients with angina and NO-CAD, thus questioning the possibility of always identifying the correct mechanism of symptoms by these tests [30].

The fact that, in the present study, ISDN was unable to significantly improve exercise results in the few patients with positive ECG-EST independently of the results of Ach is also in keeping with this view. When considering the high sensitivity of CAS to nitrates, indeed, the substantial lack of improvement in ischemic ST changes by ISDN in the CAS-group (in our study, seven out of eight CAS patients had positive ECG-EST under ISDN effects, without any difference compared to the basal test) suggests that CAS was unlikely to be the cause of positive exercise-induced ischemia, thus also questioning its pathogenetic effects for the angina symptoms of the patients.

The fact that the ECG-EST variables had a similar lack of consistent beneficial effects by ISDN in the three groups of patients with different Ach test results, in fact, may suggest that coronary microvascular abnormalities, rather than CAS, are major causes of the abnormal ischemic-like findings recorded during the EST. Previous studies, indeed, reported no, or even detrimental, effects on the EST results of short-acting nitrates in patients with the clinical picture of cardiac syndrome X (i.e., exercise-induced angina, positive EST, angiographically normal coronary arteries), in whom CMD was shown to be involved [14,15,16].

## 5. Limitations of the Study

Some limitations of our study should be acknowledged. First, this is a single-center study with a relatively small sample size and limited statistical power; thus, our findings need confirmation in larger multicenter studies. Second, our study involves a heterogeneous population, including both patients with chronic stable angina and those presenting with a non-ST-segment elevation acute coronary syndrome, including MINOCA. Unfortunately, a separate analysis of these two subgroups in our study was precluded by the low number of patients; therefore, whether differences may exist in the EST results and EST response to nitrates between these three subgroups needs to be addressed in further appropriately designed studies. 

## 6. Conclusions

In conclusion, our data show that patients with angina syndromes with NO-CAD show largely comparable results of ECG-EST and similar poor effects of short-acting nitrates on abnormal ECG-EST results, independently of the results of Ach provocation testing. On the whole, these findings suggest caution in attributing to the results of Ach test a definite causal role for the clinical syndrome in individual patients.

## Figures and Tables

**Table 1:** Main clinical characteristics of patients.

	CAS-Group (*n* = 40)	CMVS-Group (*n* = 14)	NEG-Group (*n* = 27)	*p*
Age (years)	60 + 10	61 + 9	56 + 13	0.23
Sex (M/F)	16/24	4/10	16/11	0.13
*Cardiovascular risk factors*				
Family history of CVD	17 (43%)	5 (36%)	14 (52%)	0.58
Hypertension	23 (58%)	11 (79%)	13 (48%)	0.17
Active smoking	11 (28%)	4 (29%)	5 (19%)	0.66
Hypercholesterolemia	28 (70%)	9 (64%)	15 (56%)	0.48
Diabetes	7 (18%)	2 (14%)	3 (11%)	0.77
*Clinical presentation*				
Stable angina	15 (38%)	5 (36%)	14 (52%)	0.44
Unstable angina	25 (63%)	9 (64%)	13 (48%)	0.44
MINOCA	7 (18%)	4 (29%)	3 (11%)	0.47
*Drug therapy*				
Beta-blockers	5 (13%)	4 (29%)	10 (37%)	0.01
Ca^2+^ channel blockers	38 (95%)	10 (71%)	7 (26%)	<0.0001
ACE-inhibitors/ARBs	18 (45%)	6 (43%)	10 (37%)	0.74
Statins	31 (78%)	5 (36%)	18 (67%)	0.02
Aspirin	24 (60%)	9 (64%)	11 (41%)	0.21

ACE = angiotensin-converting enzyme; ARB = angiotensin receptor blockers; CVD = cardiovascular disease; MINOCA = myocardial infarction with non-obstructive coronary artery disease.

**Table 2:** Results of baseline EST in the 3 groups of patients.

	CAS-Group (*n* = 40)	CMVS-Group (*n* = 14)	NEG-Group (*n* = 27)	*p*
*Rest*				
HR (bpm)	82 + 17	81 + 23	77 + 15	0.43
Systolic BP (mmHg)	126 + 15	131 + 19	125 + 12	0.49
Diastolic BP (mmHg)	81 + 11	80 + 12	80 + 8	0.98
RPP (bpm × mmHg)	10,361 + 2687	10,703 + 3286	9594 + 2154	0.36
*Peak exercise*				
HR (bpm)	152 + 23	144 + 16	156 + 17	0.24
Systolic BP (mmHg)	164 + 26	172 + 24	163 + 23	0.49
Diastolic BP (mmHg)	89 + 12	95 + 13	87 + 11	0.11
RPP (bpm × mmHg)	24,828 + 4618	24,848 + 4159	25,283 + 3806	0.90
Duration of exercise (s)	464 + 206	463 + 154	570 + 168	0.06
STD > 1 mm	8 (22%)	7 (50%)	6 (22%)	0.076
Angina	4 (10%)	4 (28%)	2 (7%)	0.08

BP = blood pressure; HR = heart rate; RPP = rate-pressure product; STD = ST segment depression.

**Table 3:** Results of B-EST and N-EST in the 3 groups of patients with a positive B-EST.

		CAS-Group (*n* = 8)	CMVS-Group (*n* = 6)	NEG-Group (*n* = 5)	*p*
*Rest*					
HR (bpm)	B-EST	81 + 13	88 + 34	74 + 14	0.58
	N-EST	81 + 11	75 + 10	73 + 11	0.43
Systolic BP (mmHg)	B-EST	124 + 15	133 + 22	123 + 8	0.48
	N-EST	126 + 9	131 + 15	118 + 4	0.16
Diastolic BP (mmHg)	B-EST	81 + 4	80 + 8	80 + 10	0.92
	N-EST	79 + 4	81 + 5	82 + 4	0.30
RPP (bpm × mmHg)	B-EST	10,096 + 2628	11,680 + 4257	9060 + 1668	0.37
	N-EST	10,226 + 1790	9968 + 2470	8670 + 1519	0.38
*Peak exercise*					
HR (bpm)	B-EST	148 + 14	140 + 13	163 + 16	0.06
	N-EST	146 + 19	146 + 7	152 + 14	0.74
Systolic BP (mmHg)	B-EST	170 + 23	180 + 30	164 + 17	0.54
	N-EST	164 + 21	175 + 32	152 + 8	0.29
Diastolic BP (mmHg)	B-EST	87 + 7	84 + 11	93 + 4	0.08
	N-EST	83 + 5	86 + 6	93 + 6	0.003
RPP (bpm × mmHg)	B-EST	25,307 + 4903	25,412 + 5497	26,512 + 1365	0.88
	N-EST	24,089 + 5324	25,593 + 5065	23,078 + 2234	0.67
EST duration (s)	B-EST	489 + 250	394 + 102	642 + 122	0.12
	N-EST	471 + 144	438 + 67	620 + 138	0.06
Positive N-EST		7 (87.5%)	4 (66.7%)	4 (80%)	0.64
Maximal STD (mm)	B-EST	1.9 + 1.2	1.1 + 0.2	1.8 + 0.3	0.18
	N-EST	1.3 + 0.9	0.7 + 0.5	1.6 + 0.7	0.12
Angina	B-EST	1 (14%)	3 (25%)	2 (22%)	0.86
	N-EST	0 (0%)	4 (33%)	2 (22%)	0.23
*1 mm STD*					
HR (bpm)	B-EST	135 + 16	139 + 13	138 + 20	0.88
	N-EST	131 + 21	146 + 7	129 + 18	0.20
Systolic BP (mmHg)	B-EST	161 + 19	177 + 31	150 + 14	0.17
	N-EST	154 + 17	173 + 33	144 + 15	0.12
Diastolic BP (mmHg)	B-EST	84 + 5	85 + 10	92 + 6	0.10
	N-EST	83 + 5	84 + 5	91 + 6	0.008
RPP (bpm × mmHg)	B-EST	21,665 + 3000	24,715 + 5857	20,544 + 2389	0.22
	N-EST	20,071 + 3981	25,297 + 5150	18,540 + 2570	0.03
Time to 1 mm (s)	B-EST	364 + 243	378 + 108	479 + 150	0.54
	N-EST	404 + 254	419 + 41	496 + 201	0.70

BP = blood pressure; HR = heart rate; RPP = rate-pressure product; STD = ST depression.

## Data Availability

The data presented in this study are available upon request from the corresponding author.

## References

[1] Patel M.R., Peterson E.D., Dai D., Brennan J.M., Redberg R.F., Anderson H.V., Brindis R.G., Douglas P.S. (2010). Low diagnostic yield of elective coronary angiography. N. Engl. J. Med..

[2] Gehrie E.R., Reynolds H.R., Chen A.Y., Neelon B.H., Roe M.T., Gibler W.B., Ohman E.M., Newby L.K., Peterson E.D., Hochman J.S. (2009). Characterization and outcomes of women and men with non-ST-segment elevation myocardial infarction and nonobstructive coronary artery disease: Results from the Can Rapid Risk Stratification of Unstable Angina Patients Suppress Adverse Outcomes with Early Implementation of the ACC/AHA Guidelines (CRUSADE) quality improvement initiative. Am. Heart J..

[3] Lanza G.A., Morrone D., Pizzi C., Tritto I., Bergamaschi L., De Vita A., Villano A., Crea F. (2021). Diagnostic approach for coronary microvascular dysfunction in patients with chest pain and no obstructive coronary artery disease. Trends Cardiovasc. Med..

[4] Ong P., Athanasiadis A., Borgulya G., Vokshi I., Bastiaenen R., Kubik S., Hill S., Schäufele T., Mahrholdt H., Kaski J.C. (2014). Clinical usefulness, angiographic characteristics, and safety evaluation of intracoronary acetylcholine provocation testing among 921 consecutive white patients with unobstructed coronary arteries. Circulation.

[5] A Montone R., Niccoli G., Fracassi F., Russo M., Gurgoglione F., Cammà G., A Lanza G., Crea F. (2018). Patients with acute myocardial infarction and non-obstructive coronary arteries: Safety and prognostic relevance of invasive coronary provocative tests. Eur. Heart J..

[6] Ford T.J., Stanley B., Good R., Rocchiccioli P., McEntegart M., Watkins S., Eteiba H., Shaukat A., Lindsay M., Robertson K. (2018). Stratified Medical Therapy Using Invasive Coronary Function Testing in Angina: The CorMicA Trial. J. Am. Coll. Cardiol..

[7] Chauhan A., Mullins P.A., Taylor G., Petch M.C., Schofield P.M. (1997). Both endothelium-dependent and endothelium-independent function is impaired in patients with angina pectoris and normal coronary angiograms. Eur. Heart J..

[8] Bøttcher M., Bøtker H.E., Sonne H., Nielsen T.T., Czernin J. (1999). Endotheliumdependent and independent perfusion reserve and the effect of L-arginine on myocardial perfusion in patients with syndrome X. Circulation.

[9] Lanza G.A., Buffon A., Sestito A., Natale L., Sgueglia G.A., Galiuto L., Infusino F., Mariani L., Centola A., Crea F. (2008). Relation between stress-induced myocardial perfusion defects on cardiovascular magnetic resonance and coronary microvascular dysfunction in patients with cardiac syndrome X. J. Am. Coll. Cardiol..

[10] Pupita G., Kaski J.C., Galassi A.R., Gavrielides S., Crea F., Maseri A. (1990). Similar time course of ST depression during and after exercise in patients with coronary artery disease and syndrome X. Am. Heart J..

[11] Kaski J.C., Rosano G.M., Collins P., Nihoyannopoulos P., Maseri A., Poole-Wilson P.A. (1995). Cardiac syndrome X: Clinical characteristics and left ventricular function: Long-term follow-up study. J. Am. Coll. Cardiol..

[12] Ong P., Athanasiadis A., Hill S., Schäufele T., Mahrholdt H., Sechtem U. (2014). Coronary microvascular dysfunction assessed by intracoronary acetylcholine provocation testing is a frequent cause of ischemia and angina in patients with exercise-induced electrocardiographic changes and unobstructed coronary arteries. Clin. Cardiol..

[13] Beltrame J.F. (2022). Management of vasospastic angina. Heart.

[14] Lanza G.A., Manzoli A., Bia E., Crea F., Maseri A. (1994). Acute effects of nitrates on exercise testing in patients with syndrome X. Circulation.

[15] Radice M., Giudici V., Marinelli G. (1995). Long-term follow-up in patients with positive exercise test and angiographically normal coronary arteries (syndrome X). Am. J. Cardiol..

[16] Russo G., Di Franco A., Lamendola P., Tarzia P., Nerla R., Stazi A., Villano A., Sestito A., Lanza G.A., Crea F. (2013). Lack of effect of nitrates on exercise stress test results in patients with microvascular angina. Cardiovasc. Drugs Ther..

[17] Knuuti J., Wijns W., Saraste A., Capodanno D., Barbato E., Funck-Brentano C., Prescott E., Storey R.F., Deaton C., Cuisset T. (2020). ESC Scientific Document Group. 2019 ESC Guidelines for the diagnosis and management of chronic coronary syndromes. Eur. Heart J..

[18] NICE Guidelines Recent-Onset Chest Pain of Suspected Cardiac Origin: Assessment and Diagnosis. Clinical Guideline [CG95] Published: 24 March 2010 Last updated: 30 November 2016. https://www.nice.org.uk/guidance/cg95.

[19] Knuuti J., Ballo H., Juarez-Orozco L.E., Saraste A., Kolh P., Rutjes A.W.S., Jüni P., Windecker S., Bax J.J., Wijns W. (2018). The performance of non-invasive tests to rule-in and rule-out significant coronary artery stenosis in patients with stable angina: A meta-analysis focused on post-test disease probability. Eur. Heart J..

[20] Rudzinski P.N., Kruk M., Debski M., Demkow M., Kepka C. (2023). Can the Application of Fractional Flow Reserve Computed Tomography in High-risk Patients with Chronic Coronary Syndrome Obviate Downstream Diagnostic Invasive Coronary Procedures?. J. Thorac. Imaging.

[21] Tang C.X., Qiao H.Y., Zhang X.L., Di Jiang M., Schoepf U.J., Rudziński P.N., Giovagnoli D.P., Lu M.J., Li J.H., Wang Y.N. (2022). Functional CAD-RADS using FFRCT on therapeutic management and prognosis in patients with coronary artery disease. Eur. Radiol..

[22] Sinha A., Dutta U., Demir O.M., De Silva K., Ellis H., Belford S., Ogden M., Wa M.L.K., Morgan H.P., Shah A.M. (2024). Rethinking False Positive Exercise Electrocardiographic Stress Tests by Assessing Coronary Microvascular Function. J. Am. Coll. Cardiol..

[23] Cannon R.O., Epstein S.E. (1988). “Microvascular angina” as a cause of chest pain with angiographically normal coronary arteries. Am. J. Cardiol..

[24] Lanza G.A., Crea F. (2010). Primary coronary microvascular dysfunction: Clinical presentation, pathophysiology, and management. Circulation.

[25] Ong P., Athanasiadis A., Borgulya G., Mahrholdt H., Kaski J.C., Sechtem U. (2012). High prevalence of a pathological response to acetylcholine testing in patients with stable angina pectoris and unobstructed coronary arteries. The ACOVA Study (Abnormal COronary VAsomotion in patients with stable angina and unobstructed coronary arteries). J. Am. Coll. Cardiol..

[26] Ohba K., Sugiyama S., Sumida H., Nozaki T., Matsubara J., Matsuzawa Y., Konishi M., Akiyama E., Kurokawa H., Maeda H. (2012). Microvascular coronary artery spasm presents distinctive clinical features with endothelial dysfunction as nonobstructive coronary artery disease. J. Am. Heart Assoc..

[27] Pirozzolo G., Seitz A., Athanasiadis A., Bekeredjian R., Sechtem U., Ong P. (2020). Microvascular spasm in non-ST-segment elevation myocardial infarction without culprit lesion (MINOCA). Clin. Res. Cardiol..

[28] Konst R.E., Damman P., Pellegrini D., Hartzema-Meijer M.J., van Uden B.J., Jansen T.P., Brandsma J., Vart P., Gehlmann H., Maas A.H. (2021). Vasomotor dysfunction in patients with angina and nonobstructive coronary artery disease is dominated by vasospasm. Int. J. Cardiol..

[29] Jansen T.P.J., Konst R.E., de Vos A., Paradies V., Teerenstra S., van den Oord S.C.H., Dimitriu-Leen A., Maas A.H.E.M., Smits P.C., Damman P. (2022). Efficacy of Diltiazem to Improve Coronary Vasomotor Dysfunction in ANOCA: The EDIT-CMD Randomized Clinical Trial. JACC Cardiovasc. Imaging.

[30] Sidik N.P., Stanley B., Sykes R., Morrow A.J., Bradley C.P., McDermott M., Ford T.J., Roditi G., Hargreaves A., Stobo D. (2024). Invasive Endotyping in Patients with Angina and No Obstructive Coronary Artery Disease: A Randomized Controlled Trial. Circulation.

